# Supplementary material for: January 2016 extensive summer melt in West Antarctica favoured by strong El Niño
Source: Nat Commun. 2017 Jun 15;8:15799. doi: 10.1038/ncomms15799 (PMC5481731; doi:10.1038/ncomms15799)
Supplement: Supplementary Information — Supplementary Figures, Supplementary Tables and Supplementary References [file ncomms15799-s1.pdf]

Supplementary Table 1. Contingency table tallying the respective counts of positive and negative surface melt index anomalies in the Ross sector as a function of the signs of the Southern Annular Mode (SAM) Index and of the Equatorial Southern Oscillation Index (SOI).

	+SOI		Neutral SOI		-SOI		Row totals
	Melt+	Melt-	Melt+	Melt-	Melt+	Melt-	
**+SAM**	1 (0.76)	6 (3.02)	1 (1.13)	5 (4.92)	1 (2.27)	0 (1.89)	14
**Neutral SAM**	0 (0.54)	1 (2.16)	0 (0.81)	6 (3.51)	1 (1.62)	2 (1.35)	10
**-SAM**	1 (0.70)	1 (2.81)	2 (1.05)	2 (4.57)	4 (2.11)	3 (1.75)	13
**Column totals**	2	8	3	13	6	5	37

The three time series used to generate the table (observed surface melt index, SAM Index, and Equatorial SOI) are standardized with respect to the 1980–2016 period. "Melt+" and "Melt-" denote positive and negative melt index anomalies, respectively. The positive, neutral, and negative phases of the SAM Index and SOI are defined based on ± 0.5 standard deviation of the two indices. Expected counts (which assume independence between melt and the two indices) are given in parentheses. Data from the austral summer of 1987–1988 are excluded from the calculations because of excessive gaps in the melt record. Owing to the low observed counts (< 5), significance tests cannot be performed on the relationships between the different variables.

Supplementary Table 2. Linear regression coefficients of the form *y = a · x + b* relating the brightness temperatures from the SMMR, SSM/I (F-8, F-11, F-13), and SSMIS (F-17) sensors.

Dependent variable (*y*)	Slope (*a*)	Independent variable (*x*)	Intercept (*b*)	Reference
Tb_{SMMR}	0.94	Tb_{F8}	2.62	Ref. 1
Tb_{F8}	1.008	Tb_{F11}	-1.17	Ref. 2
Tb_{F13}	0.988	Tb_{F11}	1.554	Ref. 3
Tb_{F17}	0.978	Tb_{F13}	1.207	This study

The regression coefficients are listed *as published* in the literature or (in the case of the F-13 to F-17 relationship) as derived in this study. In Supplementary Table 3, we provide the regression coefficients used to calibrate SMMR and SSM/I (F-8, F-11, F-13) brightness temperatures with respect to SSMIS F-17 brightness temperatures.

Supplementary Table 3. Linear regression coefficients of the form $y = a \cdot x + b$ relating the brightness temperatures from the SMMR and SSM/I (F-8, F-11, F-13) sensors to the brightness temperatures from SSMIS F-17.

Dependent variable (y)	Slope (a)	Independent variable (x)	Intercept (b)
Tb_{SMMR_corr}	1.020	Tb_{SMMR}	1.177
Tb_{F8_corr}	0.959	Tb_{F8}	3.848
Tb_{F11_corr}	0.966	Tb_{F11}	2.727
Tb_{F13_corr}	0.978	Tb_{F13}	1.207

These coefficients are directly inferred from linear combinations of those listed in Supplementary Table 2. The independent variable (x) corresponds to the original brightness temperatures provided by the National Snow and Ice Data Center. The dependent variable (y) corresponds to the corrected brightness temperatures.

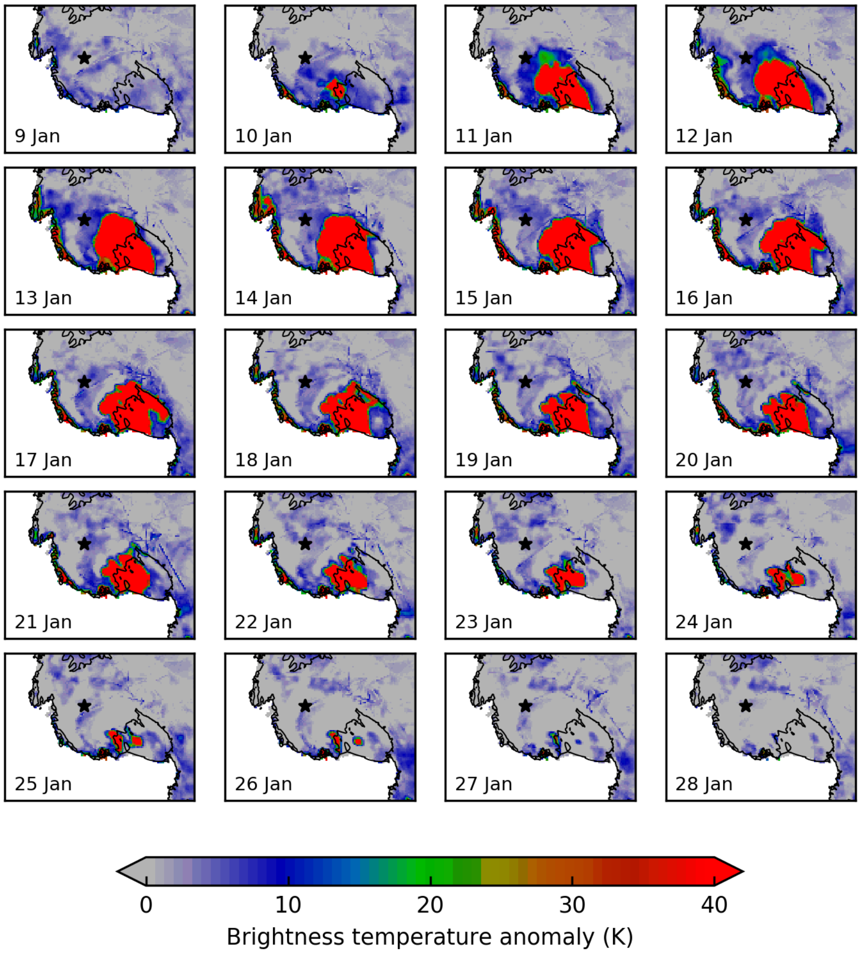

Supplementary Figure 1 | Daily anomalies in brightness temperatures over West Antarctica showing the unfolding of the January 2016 melt event. The temperatures are horizontally polarized 19GHz brightness temperatures measured by the SSMIS sensor onboard the DMSP F17 satellite. The anomalies are calculated with respect to the mean of the nine days immediately preceding the melt event (1–9 January 2016). Our algorithm identifies surface melt whenever the brightness temperature change exceeds 30 K (see Methods). The black star denotes the location of the WAIS Divide field camp.

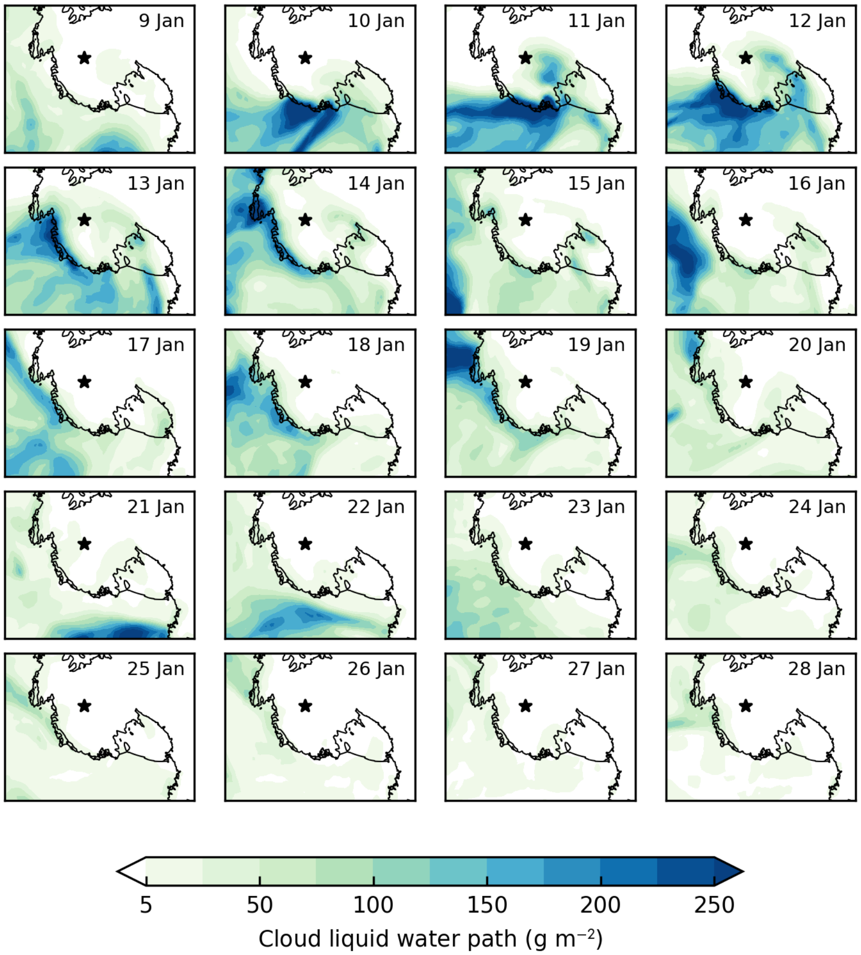

Supplementary Figure 2 | Maps of daily mean cloud liquid water path (CLWP) estimated by ERA-Interim throughout the January 2016 melt event. The black star denotes the location of the WAIS Divide field camp.

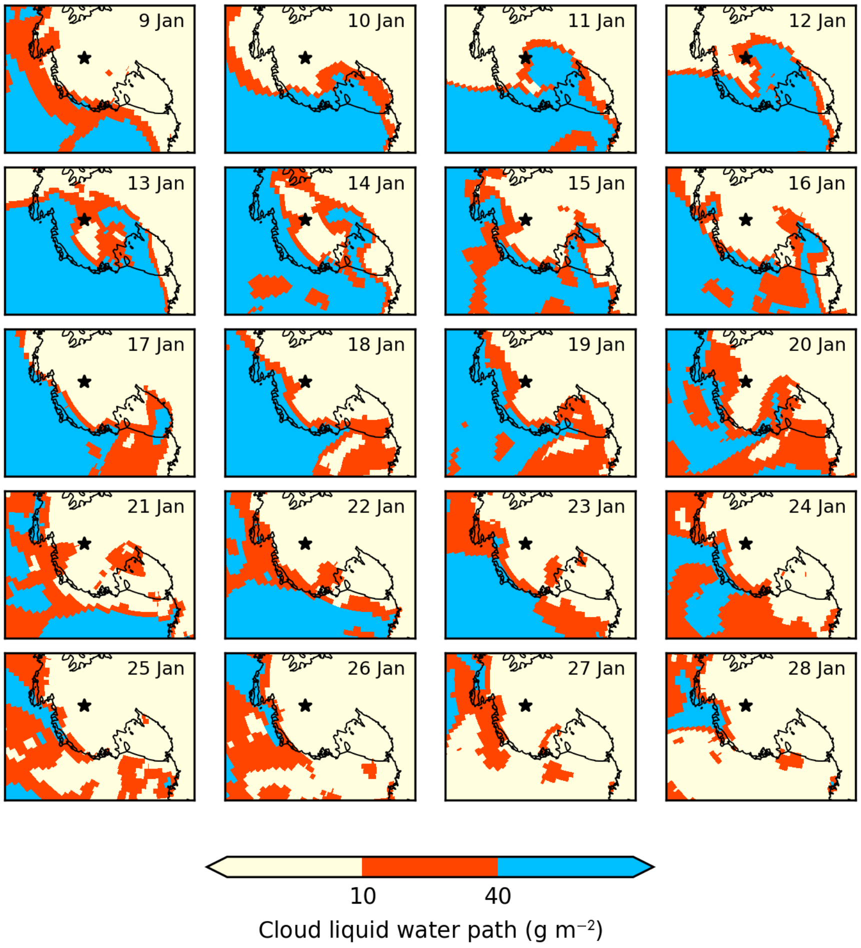

Supplementary Figure 3 | Maps of daily mean cloud liquid water path (CLWP) estimated by ERA-Interim throughout the January 2016 melt event and highlighting the range of CLWP values (10–40 g m^{-2}) relevant for the radiative enhancement mechanism described in ref. 4. The CLWP data are the same as those used for Supplementary Fig. 2. Only the color scale is different. The black star denotes the location of the WAIS Divide field camp.

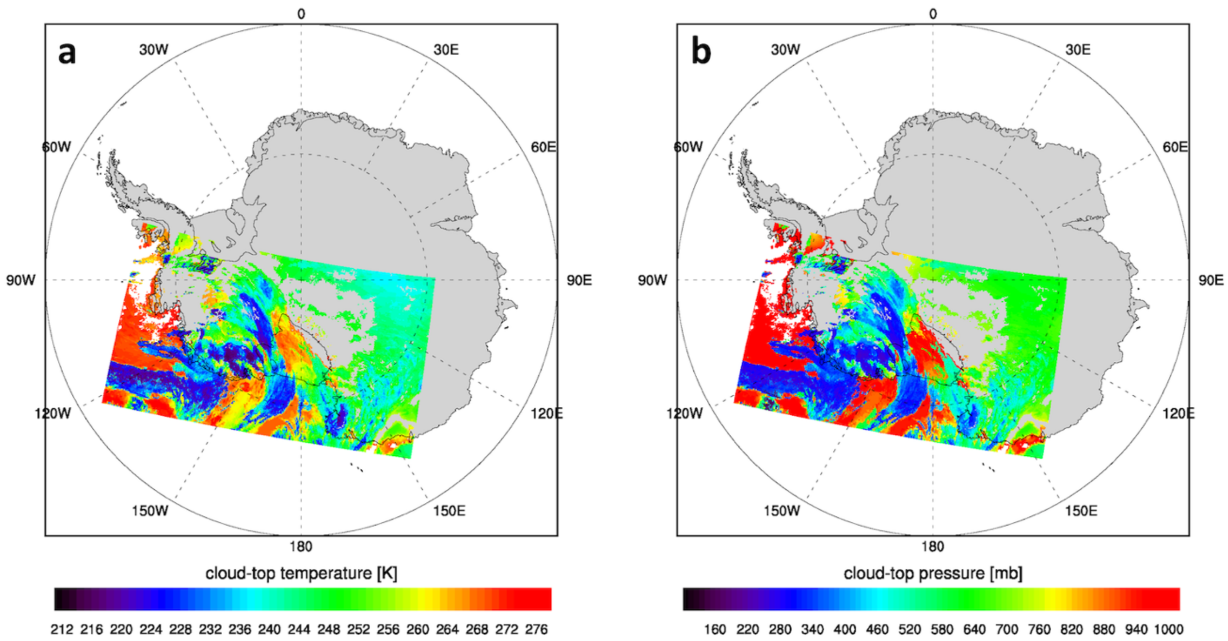

Supplementary Figure 4 | Estimates of cloud-top temperature and pressure from MODIS/Aqua for 06:50–06:55 UTC on 11 January 2016. The data are from two MODIS/Aqua Level 2 5-Min Swath 1 km and 5 km Cloud Products (MYD06). These images highlight the presence of warm (i.e., liquid-bearing) low-level clouds over the Ross Ice Shelf.

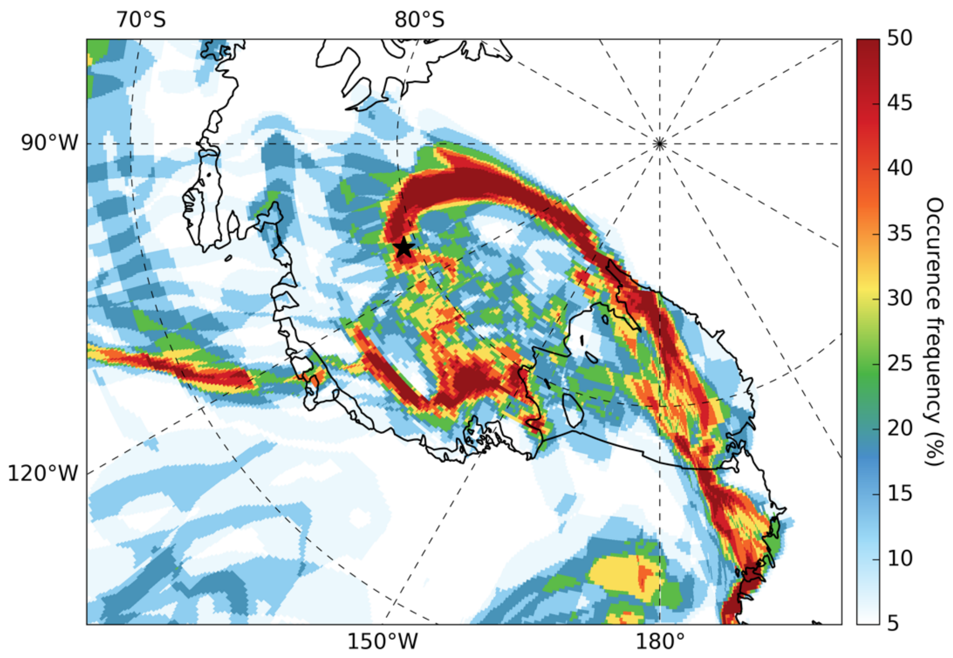

Supplementary Figure 5 | Occurrence frequency of cloud liquid water path (CLWP) within 10–40 g m^{-2} during 10–13 January 2016 based on ERA-Interim. The black star denotes the location of the WAIS Divide field camp.

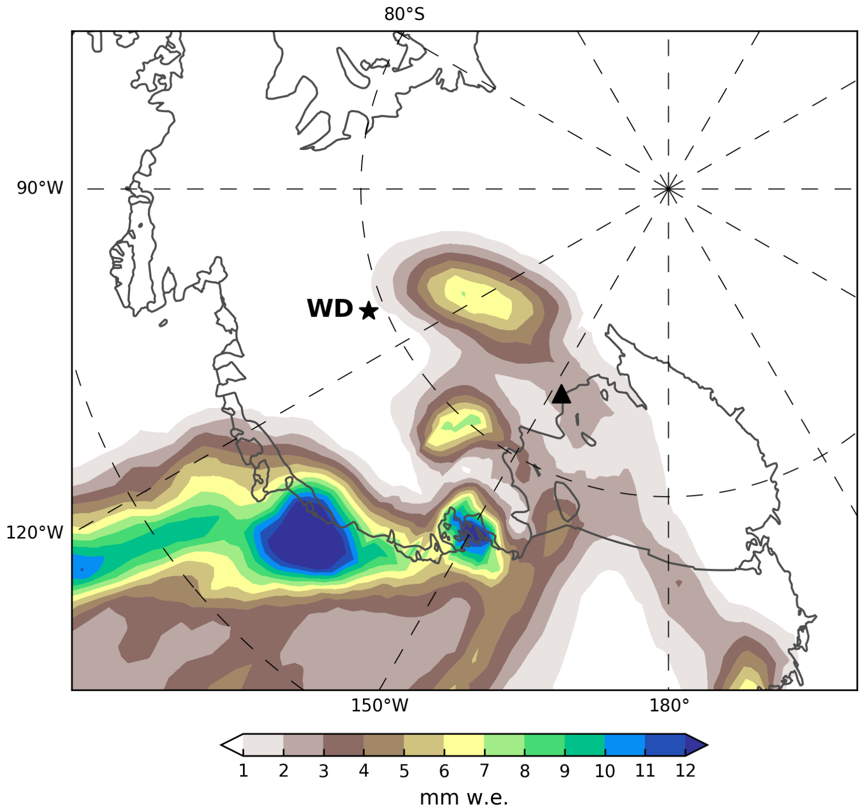

Supplementary Figure 6 | Accumulated rainfall estimates from ERA-Interim for 11–12 January 2016. The black star denotes the location of the WAIS Divide field camp. The black triangle denotes where rain was witnessed by a field party on 12 January (Dr. Huw Horgan, Victoria University of Wellington, personal communication).

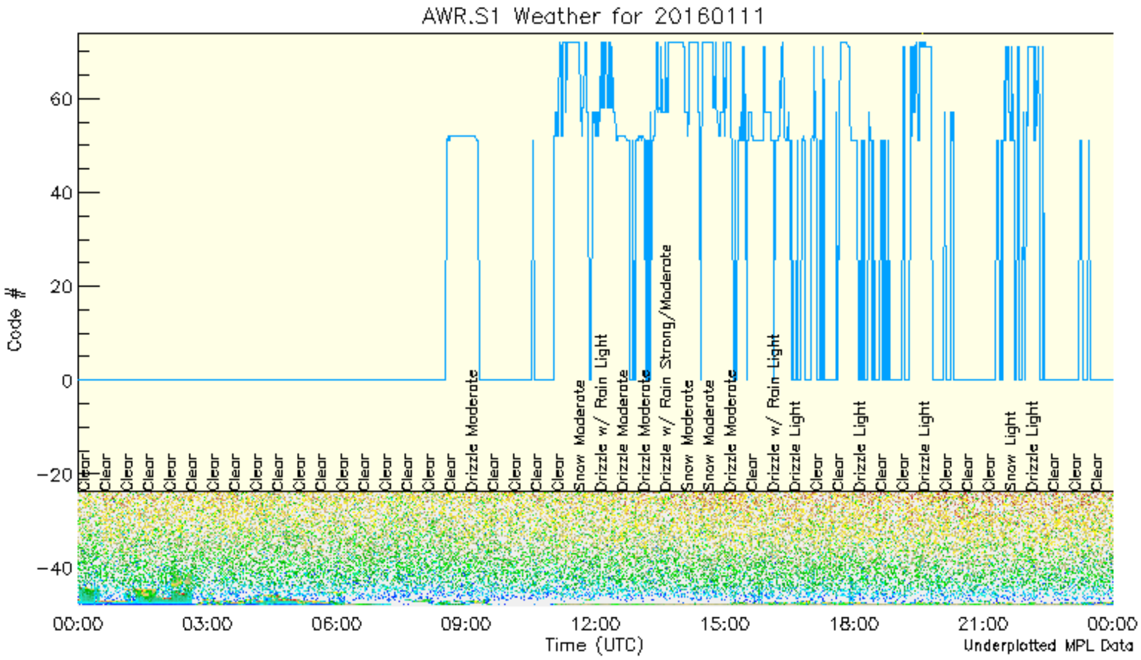

Supplementary Figure 7 | Precipitation type and intensity observed at WAIS Divide on 11 January 2016 based on measurements from a Parsivel optical disdrometer. The figure is courtesy of the ARM Program and is part of the large collection of plots publicly available on the ARM website (https://i.arm.gov > DQ Plot Browser > AWR > S1). "AWR" refers to the AWARE campaign. "S1" refers to the WAIS Divide site.

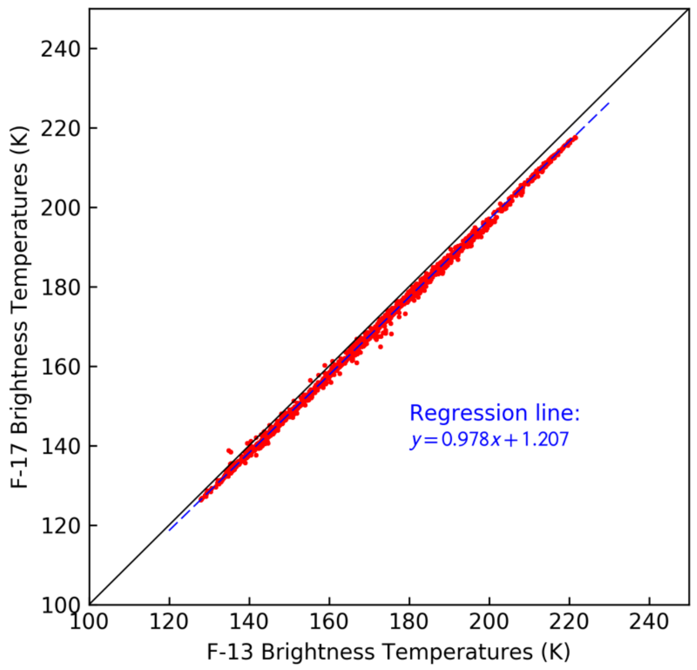

Supplementary Figure 8 | Scatter plot and regression line of daily mean brightness temperatures from SSMIS F-17 versus daily mean brightness temperatures from SSM/I F-13. The temperatures are limited to the Ross sector of West Antarctica (land points only), and to a four-month period (1 May to 30 September 2007) during the overlap period between the F-13 and F-17 missions. The May–September months are chosen for consistency with the cross-calibration between F-11 and F-13 brightness temperatures described in ref. 3.

Supplementary References

1. Jezek, K. C. *et al.* Comparison Between SMMR and SSM/I Passive Microwave Data Collected Over the Antarctic Ice Sheet. (1991).

2. Abdalati, W., Steffen, K., Otto, C. & Jezek, K. C. Comparison of brightness temperatures from SSMI instruments on the DMSP F8 and FII satellites for Antarctica and the Greenland ice sheet. *Int. J. Remote Sens.* **16,** 1223–1229 (1995).

3. Stroeve, J., Maslanik, J. & Xiaoming, L. An Intercomparison of DMSP F11- and F13-Derived Sea Ice Products. *Remote Sens. Environ.* **64,** 132–152 (1998).

4. Bennartz, R. *et al.* July 2012 Greenland melt extent enhanced by low-level liquid clouds. *Nature* **496,** 83–86 (2013).
